# Intricate predatory decisions by a mosquito-specialist spider from Malaysia

**DOI:** 10.1098/rsos.140131

**Published:** 2014-10-01

**Authors:** Robert R. Jackson, Daiqin Li, Jeremy R. W. Woon, Rosli Hashim, Fiona R. Cross

**Affiliations:** 1 School of Biological Sciences, University of Canterbury, Private Bag 4800, Christchurch, New Zealand; 2 International Centre of Insect Physiology and Ecology (ICIPE), Thomas Odhiambo Campus, PO Box 30, Mbita Point 40305, Kenya; 3 Department of Biological Sciences, National University of Singapore, 14 Science Drive 4, 117543, Singapore; 4 Centre for Behavioural Ecology and Evolution, College of Life Sciences, Hubei University, Wuhan, Hubei 430062, People's Republic of China; 5 National Parks Board, Singapore Botanic Gardens, 1 Cluny Road 259569, Singapore; 6 Faculty of Science, Institute of Biological Sciences, University of Malaya, Kuala Lumpur 50603, Malaysia

**Keywords:** mosquitoes, perception, predatory specialization, prey-choice behaviour, prey preference, Spartaeinae

## Abstract

*Paracyrba* *wanlessi* is a southeast Asian jumping spider (Salticidae) that lives in the hollow internodes of fallen bamboo and preys on the larvae, pupae and adults of mosquitoes. In contrast to *Evarcha* *culicivora*, an East African salticid that is also known for actively targeting mosquitoes as preferred prey, there was no evidence of *P*. *wanlessi* choosing mosquitoes on the basis of species, sex or diet. However, our findings show that *P*. *wanlessi* chooses mosquitoes significantly more often than a variety of other prey types, regardless of whether the prey are in or away from water, and regardless of whether the mosquitoes are adults or juveniles. Moreover, a preference for mosquito larvae, pupae and adults is expressed regardless of whether test spiders are maintained on a diet of terrestrial or aquatic prey and regardless of whether the diet includes or excludes mosquitoes. Congruence of an environmental factor (in water versus away from water) with prey type (aquatic versus terrestrial mosquitoes) appeared to be important and yet, even when the prey were in the incongruent environment, *P*. *wanlessi* continued to choose mosquitoes more often than other prey.

## 2. Introduction

Among ecologists, there has been a long-standing interest in predatory specialization [1,2], especially when the prey in question is an agricultural pest or a human–disease vector [3–6]. However, the terms ‘specialized’ and ‘generalized’ can be seriously misleading when used to specify the range of prey in a predator's natural diet. When discussing a predator's diet, it is more instructive to acknowledge a stenophagy–euryphagy continuum, with ‘stenophagy’ referring to a narrow range of prey types in a natural diet and ‘euryphagy’ referring to a wide range. It is more appropriate to use the term ‘specialization’ in the context of specifying the level to which a predator has become especially well adapted to particular prey types [7]. Specialized adaptations may include the predator's prey-choice behaviour and a cognitive attribute called ‘preference’ (e.g. [8]).

As illustrated by recent research on jumping spiders (Salticidae) [9], answering questions about stenophagy and euryphagy is an ecological exercise that has no direct implications related to whether a predator makes active choices based on its differential motivation to attack, kill and eat different types of prey (i.e. preference). This means that conclusions pertaining to prey-choice behaviour and preferences must be supported by rigorous evidence from appropriately designed experiments [8]. Perhaps all salticids include a wide taxonomic range of prey in their natural diets and yet many of the species in this large spider family [10] are also known to express remarkably specific preferences for particular types of prey [11]. By expressing distinctive specialized preferences despite also eating many other prey, these salticids illustrate that ‘specialized’ does not mean ‘restricted’.

With their complex eyes and their ability to see with exceptional spatial acuity [12,13], salticids are especially suitable subjects for prey-choice experiments because they readily reveal their choices when presented with lures made from dead prey mounted in a lifelike posture and when presented with virtual prey rendered by computer animation [14,15]. This in turn means that, when designing experiments, we can avoid the potentially confounding variables that arise when using living prey.

There are more than 5000 species in the family Salticidae, with the majority belonging to a group known as the ‘salticoids’ [16]. Active choice of ants as preferred prey (‘myrmecophagy’: [17]) has been demonstrated experimentally for a sizeable minority of the salticoid species and, for one salticoid [18,19], *Myrmarachne* *melanotarsa* (Wesolowska & Salm, 2002), there is experimental evidence of araneophagy (i.e. spider-specific prey-capture behaviour and active choice of spiders as prey). However, striking examples of araneophagy are characteristic of the Spartaeinae [20], a non-salticoid subfamily largely confined to tropical Africa, Asia and Australasia [21].

Spiders and ants tend to be dangerous prey for a salticid [22] and it has been suggested that this element of risk may favour an especially intricate expression of predatory specialization [11]. *Evarcha* *culicivora* Wesolowska & Jackson, 2003, however, appears to be an exception. This East African salticoid is highly specialized as a predator of a prey type that is not particularly dangerous. It feeds indirectly on vertebrate blood by actively choosing blood-carrying female mosquitoes as preferred prey [23]. With mosquitoes being notorious vectors of a variety of human diseases [24,25], including yellow fever, dengue and especially malaria, finding a predator that singles out mosquitoes as preferred prey is of exceptional interest [26]. With this salticid, we also see remarkable fine tuning of preference because *E*. *culicivora* also singles out *Anopheles* as its preferred mosquitoes [27], this being the mosquito genus to which all human malaria vectors belong [28].

However, previous research on *Paracyrba* *wanlessi* Żabka & Kovac, 1996, a spartaeine salticid from peninsular Malaysia, suggests that there is at least one other predator that has, by convergent evolution, become specialized at preying on mosquitoes. This salticid's habitat is the hollow internodes (culms) of fallen bamboo [29]. Water and light enter the internodes through holes made by insects and decay, and *P*. *wanlessi*'s natural diet is biased towards aquatic insects, especially towards mosquito larvae found in the phytotelma (small bodies of accumulated rainwater) inside the culms. However, *P*. *wanlessi* also eats adult mosquitoes [30]. As there has, to our knowledge, been no subsequently published research on this remarkable salticid's predatory strategy, we made experimental determination of *P*. *wanlessi*'s prey-choice behaviour our objective.

The question of whether this salticid singles out mosquitoes as preferred prey is overly simplistic because larval, pupal and adult mosquitoes are distinctively different animals, with only the adults living away from water. Substantial preliminary work was necessary because encounters with prey in water and away from water are considerably different scenarios, and we could not simply rely on using the same experimental procedures known to be effective at revealing the prey-choice behaviour of salticids that target only terrestrial prey (e.g. [31]). Incidental to this preliminary work, we acquired important qualitative information that we summarize here because it is critical for understanding our experimental methods and our findings.

One of our goals was to determine whether *P*. *wanlessi* bases prey-choice decisions on discerning the species, sex and diet of the mosquitoes it encounters. Another goal was to determine whether the preferences expressed by *P*. *wanlessi* are innate. By rearing different individuals on different diets, our objective was to determine whether *P*. *wanlessi*'s preferences were influenced by prior experience with particular prey types.

## 3. Material and methods

### 3.1 General

We collected *P*. *wanlessi* at the Ulu Gombak Field Studies Centre of the University of Malaya in Selangor Darul Ehsan (West Malaysia) by opening fallen bamboo and finding *P*. *wanlessi* in the hollow internodes [29]. Using eggs of the field-collected spiders, we then established laboratory cultures and used spiders from the second and later generations as test spiders in our experiments. The findings we report here, based on exceptionally large sample sizes, all came from experiments carried out in the Kenya laboratory over a 7 year period.

On the whole, the laboratory-rearing methods that we adopted corresponded to those that have become standard for salticid research in our laboratories (e.g. [32]). Only essential details are provided here. The laboratory photoperiod was 12 L : 12 D, with lights coming on at 07.00. Our test spiders were adults and juveniles, with all adults being female because we knew, from preliminary testing, that adult males of *P*. *wanlessi* were less responsive to prey in our experiments. For standardization, each test spider was 5–10 mm in body length. The adult females that we used had reached maturity 5–10 days before testing and none had mated. The juveniles that we used had moulted at least 5 days before testing and none moulted again in fewer than 5 days after testing.

In our experiments, we used an assortment of aquatic prey (i.e. arthropods that normally live in or on water; e.g. mosquito larvae) and terrestrial prey (i.e. arthropods that normally spend most of their time neither in nor on water; e.g. adult mosquitoes) (table 1). Mosquitoes (*Aedes aegypti* (culicine), *Anopheles* *gambiae* (anopheline) and *Culex* *quinquefasciatus*(culicine); hereafter referred to simply as *Aedes*, *Anopheles* and *Culex*), stemborers and *E*. *culicivora* came from laboratory cultures, but all other prey were collected locally in Kenya as needed. When choosing the prey to use in experiments, a primary factor was the reliable availability of a species in sufficient numbers. This meant that, although we used a wide variety of prey species for our preliminary work, only a selection of these prey were later used in experiments.

**Table 1.** Arthropods used in maintenance diets and for making lures in prey-choice experiments.

order	family	species	common name	stages used
Coleoptera	Dytiscidae	unidentified	diving beetle	larva
Diptera	Culicidae	*Aedes aegypti* (Linnaeus in Hasselquist, 1762)	culicine mosquito	adult and larva
Diptera	Culicidae	*Anopheles gambiaes*.*s*. (Giles, 1902)	anopheline mosquito	adult, larva and pupa
Diptera	Culicidae	*Culex quinquefasciatus* (Say, 1823)	culicine mosquito	adult, larva and pupa
Diptera	Chaoboridae	*Chaoborus* sp.	lake fly (midge)	adult
Diptera	Chironomidae	*Nilodorum brevibucca* (Kieffer, 1922)	lake fly (midge)	adult
Diptera	Tephritidae	*Ceratitis capitata* (Wiedemann, 1824)	fruit fly	adult
Ephemeroptera	Baetidae	unidentified	mayfly	adult and naiad
Heteroptera	Corixidae	unidentified	water boatman	nymph
Heteroptera	Gerridae	unidentified	water strider	nymph
Heteroptera	Mesoveliidae	unidentified	water treader	nymph
Heteroptera	Miridae	unidentified	leaf bug	nymph
Heteroptera	Naucoridae	unidentified	creeping water bug	nymph
Heteroptera	Notonectidae	unidentified	backswimmer	nymph
Lepidoptera	Pyralidae	*Chilopartellus* (Swinhoe, 1885)	stemborer	caterpillar
Orthoptera	Gryllidae	unidentified	cricket	nymph
Araneae	Hersiliidae	*Hersilius caudata* (Audouin, 1826)	tree-trunk spider	juvenile
Araneae	Lycosidae	*Pardosa messingerae*(Strand, 1916)	wolf spider	juvenile
Araneae	Nephilidae	*Nephilengys* sp.	web-building spider	juvenile
Araneae	Salticidae	*Evarcha culicivora*	jumping spider	juvenile
Araneae	Theridiidae	*Argyrodes* sp.	kleptoparasitic spider	adult

Using the specific prey that would be present in bamboo was not feasible, nor was this our goal. However, we should note that mosquitoes from the genera *Anopheles*, *Aedes* and *Culex* have been found in the internodes occupied by *P*. *wanlessi* [29]. Although *P. wanlessi*'*s* dominant prey in the field appears to be larvae of mosquitoes from the genus *Toxorhynchites*, unreliable access to this mosquito meant that we could not, realistically, use it in our experiments. Besides mosquitoes, the arthropods we used as potential prey in our experiments included other dipterans, as well as coleopterans, ephemeropterans, heteropterans, lepidopterans, orthopterans and a variety of spiders (Araneae).

The staple diet of the adult mosquitoes from our cultures was a 6% glucose solution [33] and, except when we indicate otherwise, it was these mosquitoes that we used for rearing test spiders and for making lures (see below). However, in some experiments, we used female mosquitoes that had fed on blood 4 h before being used for making a lure [23].

At random, test spiders were assigned to diet-related groups, and provisioning with prey began as soon as the spiders emerged from their egg sacs. Most spiders were assigned to either the ‘standard terrestrial diet’ (table 2) or the ‘standard aquatic diet’ (table 3). Mosquitoes were excluded from these standard diets. In some instances, spiders were maintained on a diet that excluded midges as well as mosquitoes. To test whether *P*. *wanlessi* has an innate preference for mosquitoes, we compared spiders that had been maintained on the standard terrestrial diet or the standard aquatic diet with spiders that had been maintained on one of four alternative diets that included mosquitoes (tables 3 and 4).

**Table 2.** Results from simultaneous-presentation testing of *P*. *wanlessi* with terrestrial prey in water. (Test spiders had been maintained on the standard terrestrial diet (no experience with mosquitoes or with spiders: *Chaoborus* sp.,*Nilodorum brevibucca* and *Ceratitis capitata*). Unless stated otherwise, mosquitoes had only fed on sugar (glucose solution). (*a*) Mosquitoes (adult females) and non-mosquito prey (*n*=25 for each row). (*b*) Adult female mosquitoes of different species (*n*=60 for each row). (*c*) Adult female mosquitoes belonging to the same species that had either fed on sugar alone or on blood (*n*=60).)

comparison	prey 1	prey 2	chose prey 1	chose prey 2	*χ*^2^ test of goodness of fit (null hypothesis: 50/50)
(*a*)	*Culexquinquefasciatus*^*a*^	*Pardosa messingerae*^*b*^	22	3	*χ*^2^=14.44, *p*<0.001
	*Culexquinquefasciatus*	*Argyrodes* sp.^*b*^	20	5	*χ*^2^=9.00, *p*=0.003
(*b*)	*Aedes aegypti*^*a*^	*Culexquinquefasciatus*	26	34	*χ*^2^=1.07, *p*=0.302
	*Aedes aegypti*	*Anopheles gambiae*^*c*^	30	30	*χ*^2^=0.00, *p*=1.000
	*Culexquinquefasciatus*	*Anopheles gambiae*	28	32	*χ*^2^=0.27, *p*=0.606
(*c*)	*Culexquinquefasciatus* (blood)	*Culexquinquefasciatus* (sugar)	33	27	*χ*^2^=0.60, *p*=0.439

^*a*^Culicine mosquito.

^*b*^Spider.

^*c*^Anopheline mosquito.

**Table 3.** Results from simultaneous-presentation testing of *P*. *wanlessi*that had been maintained on different diets. (Aquatic prey in water used when testing. (*a*) Culicine and anopheline mosquitoes (*n*=60 for each row). (*b*) Mosquito and non-mosquito prey (*n*=60 for each row). (Standard aquatic diet (no experience with mosquitoes or with baetids): unidentified Corixidae, Naucoridae and Notonectidae. Alternative diet 1 (experience with mosquitoes and with baetids): *C*. *quinquefasciatus* larvae and unidentified Baetidae.)

comparison	diet	prey 1	prey 2	chose prey 1	chose prey 2	*χ*^2^ test of goodness of fit (null hypothesis: 50/50)	*χ*^2^ test of independence
(*a*)	standard aquatic diet	*Culex quinquefasciatus* larva	*Anopheles gambiae* larva	36	24	*χ*^2^=2.40, *p*=0.121	*χ*^2^=0.82, *p*=0.359
	alternative diet 1			31	29	*χ*^2^=0.07, *p*=0.796	
(*b*)	standard aquatic diet	*Culex quinquefasciatus* larva	baetid naiad	41	19	*χ*^2^=8.07, *p*=0.005	*χ*^2^=2.13, *p*=0.144
	alternative diet 1			48	12	*χ*^2^=21.60, *p*<0.001

**Table 4.** Results from simultaneous-presentation testing of *P*. *wanlessi*maintained on different diets. ((*a*) Adult female anopheline and culicine mosquitoes away from water (*n*=60 for each row). (*b*) Adult female mosquito and non-mosquito terrestrial prey away from water (*n*=60 for each row). (*c*) Larval mosquito and non-mosquito aquatic prey in water (*n*=25 for each row). Standard terrestrial diet (no experience with prey used when testing): see table 2. Alternative diet 2 (experience with mosquitoes): *A. gambiae* adults, *C*. *quinquefasciatus* adults and *Ceratitis capitata*. Alternative diet 3 (experience with mosquitoes and with spiders): *A. gambiae* adults, *C*. *quinquefasciatus* adults and *Argyrodes* sp. Alternative diet 4 (experience with mosquitoes and with naucorids): *C*. *quinquefasciatus* larvae plus unidentified Corixidae, Naucoridae and Notonectidae.)

comparison	diet	prey 1	prey 2	chose prey 1	chose prey 2	*χ*^2^ test of goodness of fit (null hypothesis: 50/50)	*χ*^2^ test of independence
(*a*)	standard terrestrial diet	*Anopheles gambiae* adult	*Culex quinquefasciatus* adult	34	26	*χ*^2^=1.07, *p*=0.302	*χ*^2^=1.20, *p*=0.273
	alternative diet 2			28	32	*χ*^2^=0.27, *p*=0.606	
(*b*)	standard terrestrial diet	*Culex quinquefasciatus* adult	*Argyrodes* sp.	23	7	*χ*^2^=8.53, *p*=0.003	*χ*^2^=0.42, *p*=0.517
	alternative diet 3			25	5	*χ*^2^=13.33, *p*<0.001	
(*c*)	standard terrestrial diet	*Culex quinquefasciatus* larva	Naucorid	24	1	*χ*^2^=21.16, *p*<0.001	*χ*^2^=3.03, *p*=0.082
	alternative diet 4			20	5	*χ*^2^=9.00, *p*=0.003	

We standardized each test spider's hunger level by maintaining it on a 5-day fast immediately before testing. Otherwise, we fed each individual to satiation 3 days per week (Monday, Wednesday and Friday), with prey for individuals on a terrestrial diet being introduced directly into the spider's rearing cage. On feeding days, individuals on an aquatic diet were first transferred to a feeding cage that was similar to that used for aquatic prey-choice testing (see below), but with living prey instead of lures in the water.

### 3.2 Preliminary work

Although investigating prey-size choice was not one of our experimental objectives, we used a wide variety of prey sizes in preliminary testing and it was evident that *P*. *wanlessi* was disinclined to attack prey larger than itself. Observing predatory sequences with prey away from water was not particularly difficult, as simply putting the predator and prey together in a cage made from plastic boxes or Petri dishes worked about as well as anything else we tried. Predatory sequences usually began with *P*. *wanlessi* fixating the gaze of its front eyes on prey that was moving and no more than about 100 mm away and then, by stepping in short bursts, *P*. *wanlessi* moved closer (i.e. it suddenly stepped forward a few millimetres, paused for several seconds, stepped forward again and so forth). When about 30 mm away, *P*. *wanlessi* assumed a special posture (the ‘pre-strike posture’), with body lowered, leg IV oriented rearward and legs I–III oriented forward, all legs being highly flexed (femora of legs I-III angled almost directly back, and these legs from the patella to the tarsus extended almost directly forward). In this posture, *P*. *wanlessi* sometimes eased forward until only a few millimetres away from the prey and then, after a highly variable interval, attacked by extending legs I–III over the prey and then flexing these legs so that the prey was scooped towards the spider's chelicerae. When it attacked, the spider sometimes suddenly, by extending its two legs IV simultaneously, moved its body rapidly forward to lunge (leg IV tarsi did not leave the substrate) or to leap (leg IV tarsi lost contact with the substrate) at the prey.

Designing effective methods for staging in-water encounters was more difficult, but we succeeded when we used an apparatus in which *P*. *wanlessi* could walk more or less directly downward to the water surface. Typically from as far as 100 mm away, *P*. *wanlessi* usually first oriented to moving prey in water and then, by stepping in short bursts, moved closer to the water. If the prey was close to the surface and especially active, *P*. *wanlessi* often then ran sideways near the water, all the while staying even with the active prey. Eventually, *P*. *wanlessi* adopted the pre-strike posture and became quiescent close to, and sometimes with its forelegs touching, the water surface [30].

After attacking prey in water, *P*. *wanlessi* often failed to hold on. When this happened, the prey usually moved rapidly away and *P*. *wanlessi* usually resumed standing in the pre-strike posture. *Paracyrba* *wanlessi* attacked the prey again when it returned and these attack sequences were sometimes repeated dozens of times before *P*. *wanlessi* finally held on to the prey with its chelicerae. Comparable sequences of repeatedly attacking prey were rare when prey was away from water.

Upon capturing prey in water, *P*. *wanlessi* usually backed rapidly up the side of the chamber, dragging the prey along. Water could be seen coming off the dragged prey. When *P*. *wanlessi* eventually settled and fed, it was usually well away from the water.

### 3.3 Lures

Instead of living prey, we used lures in all experiments and considerable effort was required for making some of the lures we needed. First, we immobilized the prey with carbon dioxide, kept it in 70% ethanol for 60 min and then, after removing it from the ethanol, positioned it in lifelike posture on a cork disc (thickness 1 mm), with the diameter of the disc being small enough to minimize its visibility below the mounted prey. A small drop of sticky gum (Tanglefoot Pest Barrier) held the prey in place on the top of the disc. For preservation and for retaining the prey's specific posture, the lure was then sprayed with a clear aerosol plastic adhesive (Crystal Clear Lacquer, Atsco Australia Pty). The lure was always about half the size of the test spider.

**Figure 1.**
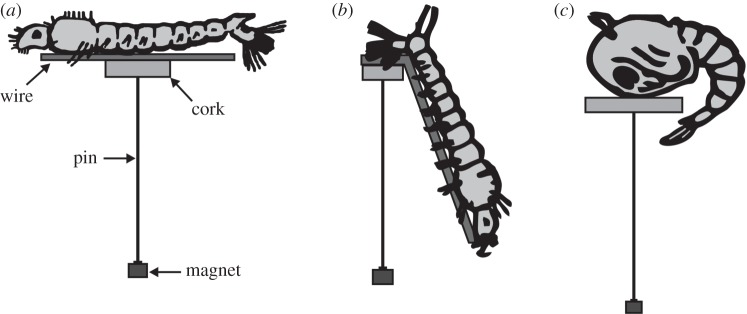
Lures made from the larvae and pupae of mosquitoes, showing how these prey types were mounted on cork discs. (*a*) *Anopheles* larva, mounted ventral side up, with head rotated 180°. (*b*) *Culex* larva, mounted in a head-down, almost-vertical posture, with the ventral surface of the larva's last abdominal segment on the top of the cork disc. (*c*) *Culex* pupa, mounted with the anterior ventral surface on top of the cork disc.

Anopheline mosquito larvae (figure 1*a*) and notonectid nymphs were mounted ventral side up, as this is the normal resting posture of these insects. All other prey were mounted dorsal side up. For prey that were especially slender and soft-bodied (caterpillars, beetle larvae and mosquito larvae), we used a piece of thin metal wire for maintaining the normal posture. Sticky gum applied to the wire held the insect to the wire and the cork disc. Caterpillars, beetle larvae and anopheline mosquito larvae were positioned horizontally on the top of the cork disc, with the disc centred midway along the length of the insect's extended body (figure 1*a*).

When feeding at the surface of the water, an anopheline larva normally rotates its head 180° [24], with the dorsal side of its head facing upward, and we replicated this posture with our lures (figure 1*a*). By contrast, it is characteristic of culicine larvae to rest in a head-down, almost-vertical posture. When in this posture, siphons at the posterior end of the culicine larva's abdomen are elevated higher than the rest of the body. We simulated this posture with our lures by positioning the ventral surface of the larva's last abdominal segment on the top of the cork disc (figure 1*b*), with the metal wire extending from this disc down at an angle of 20°. Mosquito pupae rest in a characteristic posture with the dorsal surface of the cephalothorax above and the rest of the body arched down and under. When making lures, we replicated this posture by positioning the anterior ventral surface of the pupa on the top of a cork disc (figure 1*c*), with the abdomen curling down and below the disc.

### 3.4 Experimental apparatus

**Figure 2.**
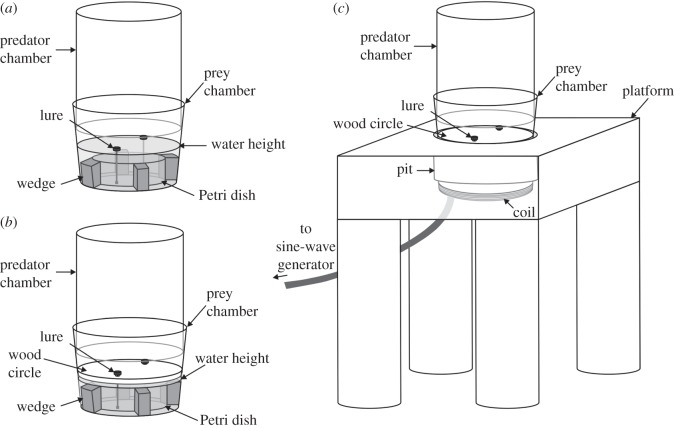
(*a*) Apparatus used in prey-choice experiments when lures were in water and (*b*,*c*) when lures were away from water. Start of test: test spider in predator chamber and walks down to prey chamber. (*c*) Prey chamber sat inside a pit on top of a wooden platform. Two lures were present at any one time, and lures were moved during the test by using a sine-wave generator connected to a coil situated underneath the platform (held with a plastic stand; not shown). End of test: test spider attacks lure.

The apparatus was made from transparent plastic and had two chambers (figure 2), a predator chamber on the top and a prey chamber on the bottom. The predator chamber was cylindrical (diameter 43 mm, height 55 mm, open at bottom, closed at top) and the prey chamber was a tapered cylinder (diameter at top 50 mm, diameter at bottom 42 mm, height 38 mm, open at the top, closed at the bottom). The combined height of these two chambers is roughly comparable to the vertical spaces of the fallen (horizontal) internodes during *P*. *wanlessi*'s encounters with prey in the field. The bamboo species in *P*. *wanlessi*'s natural habitat [29,30], *Gigantochloa scortechinii* and *G*. *ligulata*, are especially large (up to 25 m), with the internodes occupied by *P*. *wanlessi* being 80–100 mm in diameter.

A plastic Petri dish (diameter 32 mm, height 10 mm, top of the dish removed) was centred on the floor of the prey chamber and positioned upside-down so that the bottom of the dish faced the predator chamber. Four wedges (each side 5 mm long; height 10 mm), made from blue closed-cell chemically cross-linked polyolefin foam, fitted tightly between the edge of the Petri dish and the edge of the prey chamber, with the upper side of each wedge flush with the top of the Petri dish. Being evenly spaced around the Petri dish, the four wedges created four slots of equal size in the space between the perimeter of the Petri dish and the perimeter of the prey chamber. Two of the slots, opposite to each other, were empty. We put a lure in each of the other two slots. Distilled water filled the bottom of the prey chamber and, for tests with prey in water (figure 2*a*), the water level was 6 mm above the Petri dish. For tests with prey away from water (figure 2*b*), the water level in the prey chamber was only 2 mm above the Petri dish. Owing to the tightly fitting wedges, the Petri dish remained in place when the water was added.

For tests with lures in water (figure 2*a*), the cork discs floated on top of the water in the prey chamber (i.e. 6 mm above the bottom of the upside-down Petri dish). For tests with lures away from water (figure 2*b*), a circular piece of white wood (thickness 2 mm) was positioned horizontally in the prey chamber. The diameter of the wood circle was such that it fitted tightly in the prey chamber, with the bottom of the wood circle being 2 mm above the water level and the top being 6 mm above the bottom of the Petri dish. There were two holes cut in the wood circle, with the diameter of each hole being 2 mm and the closest edge of the hole being 2 mm from the edge of the wood circle. Each hole was positioned over the centre of one of the slots in which there was a lure. The sharp end of a metal pin (diameter 0.5 mm) was inserted into the bottom of each cork disc, and this pin extended through the hole, with the lure sitting on the wood above the hole.

As preliminary work showed that moving lures were more effective than stationary lures at eliciting a response from *P*. *wanlessi*, we used a coil-and-magnet system for making lures move during our experiments. A cube-shape nickel cadmium magnet (each side 1 mm long) was glued to the blunt end of the pin, distal to the lure. The length of the pin was such that the magnet was held suspended in the water 3 mm above the floor of the prey chamber. We standardized the movement of the lures during experiments by using a sine-wave generator connected to a coil (diameter 50 mm). During testing, a sine wave (5 Hz) was repeatedly pulsed on for 1 s and then turned off for 9 s. Amplitude was adjusted so that the lures moved 2 mm back and forth during each pulse.

We set the apparatus on top of a horizontal wooden platform (95×95 *mm*; round leg at each corner: height 95 mm, diameter 30 mm; wood thickness: 30 mm) (figure 2*c*). There was a pit in the centre of the platform. The depth and diameter of the pit were such that the prey chamber fitted securely inside, with the water level in the prey chamber being even with the top of the pit. The platform, including the inside of the pit, was painted white. The coil was held horizontal by a plastic stand 10 mm below the bottom surface of the platform and centred directly below the pit.

### 3.5 Experimental procedures

The test spider was confined to the predator chamber for 24 h by a plastic screw cap at the bottom of the chamber. For initiating testing, we removed the cap and pushed the predator chamber into the top of the prey chamber so that the open bottom end of the predator chamber was 25 mm from the bottom end of the prey chamber. However, we initiated testing only if the test spider was quiescent in the normal resting posture and positioned on the wall of the chamber facing down and with tarsi IV touching, or close to touching, the top of the chamber. As a prerequisite for continuing with the test, we also required that the spider remained quiescent in this posture and at this location in the chamber when the screw cap was removed from the predator chamber and then while the predator chamber was positioned in the prey chamber. It was rare that these prerequisites were not met, as *P*. *wanlessi* typically adopts this posture when at rest in these chambers, and typically remains quiescent in this posture for extended periods.

The end of a test was when the test spider attacked a lure. We aborted testing whenever 30 min elapsed without the spider attacking a lure. Attacking was always preceded by the test spider stalking the lure until close. Our definition of ‘close’ was the anterior end of the spider's body being sufficiently near the lure to permit contacting the lure by lunging (5–10 mm, depending on the size of the test spider). Our definition of ‘stalking’ was the test spider stepping, usually in short bursts, towards a lure, all the while facing the lure. Between tests, the apparatus was washed with 80% ethanol followed by distilled water, and then dried.

We adopted two testing methods (simultaneous-presentation and alternate-day). Although some details were unique to testing *P*. *wanlessi*, the underlying protocol for each of these methods was the same as in numerous previous studies from our laboratories (e.g. [31]). During simultaneous-presentation testing, two different prey types were present at the same time (i.e. there was a lure made from one prey type in one prey slot and a lure made from another prey type in the other prey slot). During alternate-day testing, each individual test spider was used in a pair of tests. On one day, two lures (one in each prey slot) made from the same prey type were present and, on the following day, two lures (one in each prey slot) made from another prey type were present. We determined at random which prey type would be used first. Simultaneous-presentation testing was used for all of the prey combinations that we considered. For determining whether findings from the two testing methods would reveal the same preferences, we adopted alternate-day testing with a selection of these prey combinations.

We also did alternate-day testing with lures in water on one day and away from water on the other day, with the condition (water or no water) adopted on the first day being determined at random. The objective of these tests was to determine whether *P*. *wanlessi* had preferences related to whether prey was in or away from water.

Consistent with our goal of investigating innate preferences, we adopted procedures that minimized opportunities to be influenced by prior experience with our testing procedures. No individual test spider was used in more than one simultaneous-presentation test or more than one pair of alternate-day tests. We also ensured that the individuals used in alternate-day testing were always different from the individuals used in simultaneous-presentation testing.

Each successful simultaneous-presentation test ended when the test spider attacked one of the two prey and the particular prey that was attacked was recorded as the test spider's choice. However, with alternate-day testing, the test spider had two opportunities to attack prey and we used the expression ‘choose’ exclusively for instances when the test spider attacked one prey type but did not attack the other prey type on the alternate-day, or attacked prey in one situation (in water or away from water) but not the other (i.e. instances of attacking both were recorded as instances of not choosing).

Data from simultaneous-presentation testing were analysed using *χ*^2^ tests of goodness-of-fit (null hypothesis: equal inclination to attack each of the two prey types). For alternate-day tests, data were analysed using *χ*^2^ McNemar tests for significance of changes. Instances of not choosing did not enter into the data analysis for either of these two types of testing. By using *χ*^2^ tests of independence, we also compared the data for test spiders that had been kept on different diets, and we applied Bonferroni corrections whenever the same datasets were used in more than one comparison (adjusted *α*=0.025).

## 4. Results and discussion

### 4.1 Preferences expressed during encounters with terrestrial prey: *Paracyrba wanlessi* compared with *Evarcha culicivora*

**Figure 3.**
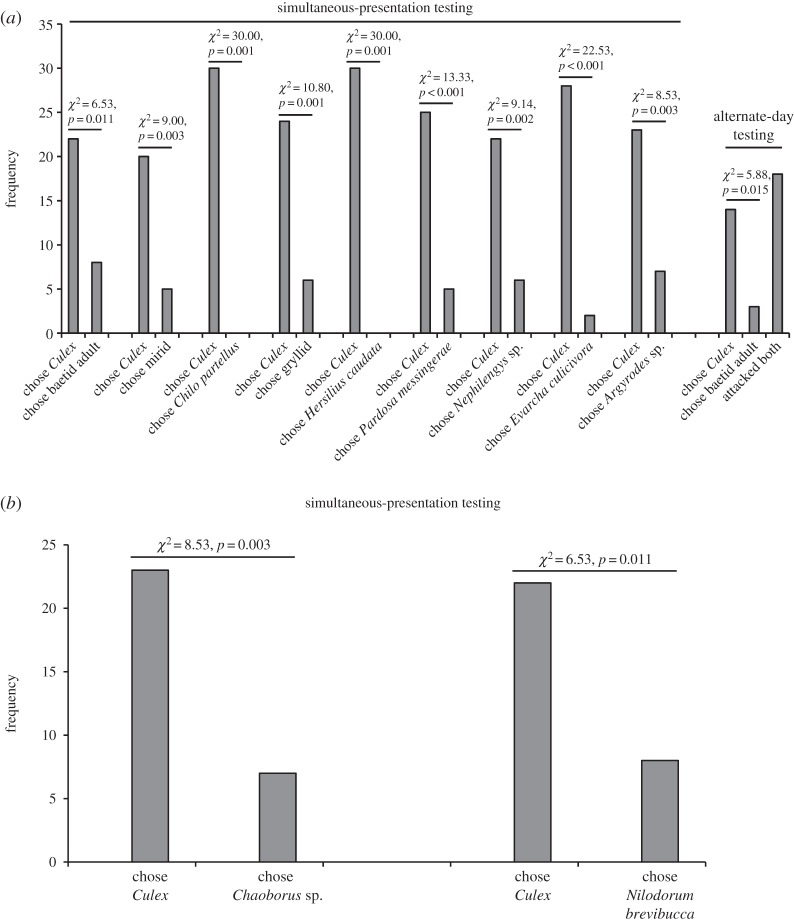
Results from testing *P*. *wanlessi* with adult females of *C*. *quinquefasciatus* and a variety of non-mosquito terrestrial prey. Prey: lures presented to test spiders away from water. Simultaneous-presentation testing: *n*=30 for each pair of prey types (except when testing with *Culex* and mirid: *n*=25). Alternate-day testing: *n*=35. (*a*) All test spiders had been maintained on the standard terrestrial diet (no experience with mosquitoes; see table 2). (*b*) All test spiders had been maintained on a diet of *C*. *capitata* and unidentified Baetidae (no experience with mosquitoes or midges).

**Figure 4.**
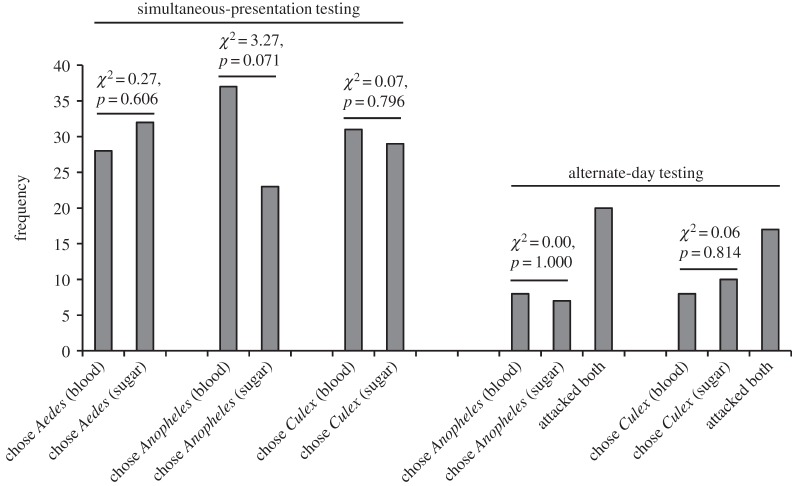
Results from testing *P*. *wanlessi* with adult females from three mosquito species, *A. aegypti* (culicine), *A. gambiae* (anopheline) and *C*. *quinquefasciatus* (culicine). Mosquitoes either had received a blood meal or had been fed only sugar (glucose solution). All test spiders had been maintained on the standard terrestrial diet (no experience with mosquitoes; see table 2). Prey: lures presented to test spiders away from water. Simultaneous-presentation testing: *n*=60 for each pair of prey types. Alternate-day testing: *n*=35 for each pair of prey types.

**Figure 5.**
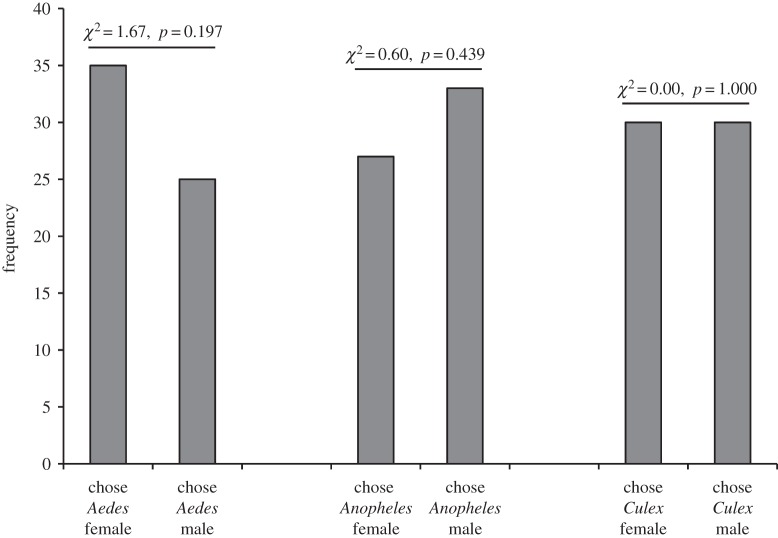
Results from simultaneous-presentation testing of *P*. *wanlessi* with adult females and adult males from three mosquito species: *A. aegypti* (culicine), *A. gambiae* (anopheline) and *C*. *quinquefasciatus* (culicine). All test spiders had been maintained on the standard terrestrial diet (no experience with mosquitoes; see table 2). Prey: lures presented to test spiders away from water. *n*=60 for each pair of prey types.

**Figure 6.**
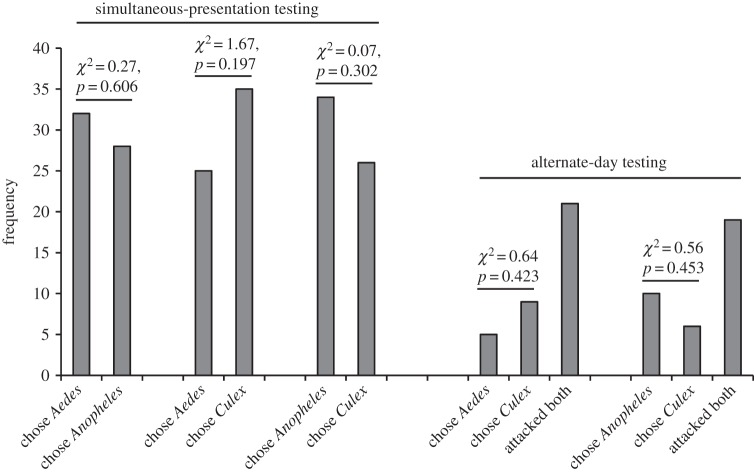
Results from testing *P*. *wanlessi* with adult females from three mosquito species: *A. aegypti* (culicine), *A. gambiae* (anopheline) and *C*. *quinquefasciatus* (culicine). All test spiders had been maintained on the standard terrestrial diet (no experience with mosquitoes; see table 2). Prey: lures presented to test spiders away from water. Simultaneous-presentation testing: *n*=60 for each pair of prey types. Alternate-day testing: *n*=35 for each pair of prey types.

In encounters away from water, *P*. *wanlessi*, like *E*. *culicivora*, chose adult mosquitoes significantly more often than a variety of other terrestrial prey types (figure 3), but there was a striking difference between these two species. Although *E*. *culicivora* feeds indirectly on vertebrate blood by expressing a preference for blood-carrying female mosquitoes as prey [23,31], we found no evidence of *P*. *wanlessi* distinguishing between mosquitoes that were or were not carrying blood (figure 4), and no evidence of *P*. *wanlessi* distinguishing between female and male mosquitoes (figure 5). Moreover, *E*. *culicivora* discriminates between anopheline and culicine mosquitoes, expressing a preference for the anophelines [27], but we found no evidence of *P*. *wanlessi* discriminating between anopheline and culicine mosquito species (figure 6). *Paracyrba* *wanlessi* expressed corresponding preferences when terrestrial prey were in water: mosquitoes were chosen significantly more often than other terrestrial prey in water (table 2, comparison *a*), but there was no evidence of *P*. *wanlessi* distinguishing between anopheline and culicine mosquitoes (table 2, comparison *b*) or between mosquitoes that had been on different diets (table 2, comparison *c*).

When we consider solely the choices made by *E*. *culicivora* and *P*. *wanlessi* when they are presented with terrestrial prey, it might be tempting to conclude that *E*. *culicivora* is more thoroughly specialized as a mosquito predator than *P*. *wanlessi*. However, other differences suggest that *P*. *wanlessi* is more thoroughly specialized than *E*. *culicivora*. For *E*. *culicivora*, only adult mosquitoes appear to be relevant because there is no evidence of *E*. *culicivora* feeding on aquatic prey. However, water is a part of *P*. *wanlessi*'s natural habitat, and this meant it was also important to investigate the preferences *P*. *wanlessi* expresses during encounters with aquatic prey in water.

### 4.2 Preferences expressed by *Paracyrba wanlessi* during encounters with aquatic prey in water

**Figure 7.**
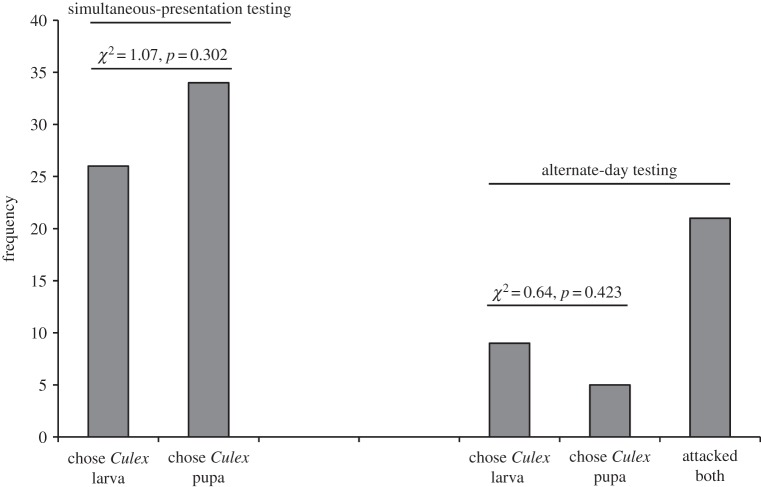
Results from testing *P*. *wanlessi* with larvae and pupae of *C*. *quinquefasciatus*. All test spiders had been maintained on the standard aquatic diet (no experience with mosquitoes; see table 3). Prey: lures presented to test spiders in water. Simultaneous-presentation testing, *n*=60; alternate-day testing, *n*=35.

**Figure 8.**
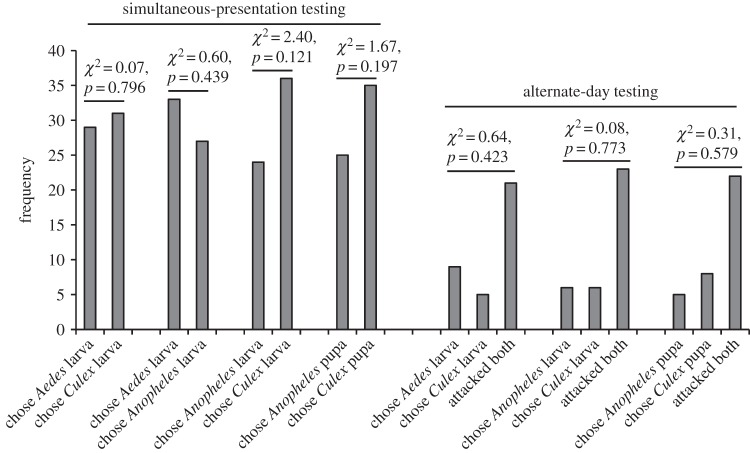
Results from testing *P*. *wanlessi* with larvae or pupae from three mosquito species: *A. aegypti* (culicine), *A. gambiae* (anopheline) and *C*. *quinquefasciatus* (culicine). All test spiders had been maintained on the standard aquatic diet (no experience with mosquitoes; see table 3). Prey: lures presented to test spiders in water. Simultaneous-presentation testing: *n*=60 for each pair of prey types. Alternate-day testing: *n*=35 for each pair of prey types.

**Figure 9.**
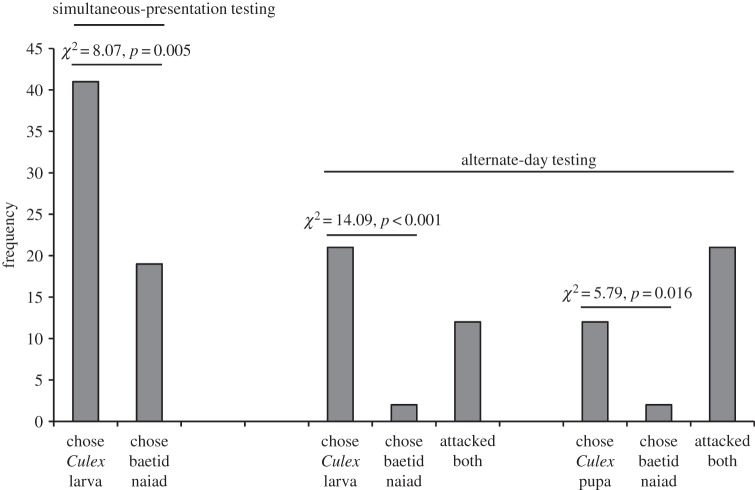
Results from testing *P*. *wanlessi* with larvae or pupae of *C*. *quinquefasciatus* and with baetid naiads. All test spiders had been maintained on the standard aquatic diet (no experience with mosquitoes or with baetids; see table 3). Prey: lures presented to test spiders in water. Simultaneous-presentation testing: *n*=60. Alternate-day testing: *n*=35 for each pair of prey types.

After being maintained on the standard aquatic diet, there was no evidence that *P*. *wanlessi* distinguished between mosquito larvae and pupae (figure 7) or between anopheline and culicine larvae or pupae (figure 8). However, *P*. *wanlessi* chose aquatic juvenile mosquitoes (larvae and pupae) significantly more often than they chose non-mosquito aquatic prey (figure 9).

### 4.3 Influence of prior diet on the expression of preferences

There was no evidence suggesting that the preferences revealed by our experiments were influenced by prior experience with different kinds of prey. Regardless of prior diet and regardless of whether prey were aquatic or terrestrial, *P*. *wanlessi* chose mosquito prey significantly more often than non-mosquito prey (tables 3 and 4). However, regardless of prior diet and regardless of whether the prey were aquatic (table 3, comparison *a*) or terrestrial (table 4, comparison *a*), there was no evidence that *P*. *wanlessi* distinguished between anopheline and culicine mosquitoes. Moreover, the data for spiders on the standard terrestrial and the standard aquatic diets (i.e. spiders that had no experience with mosquitoes) were not significantly different (tests of independence) from the data for spiders on any of the alternative diets (i.e. spiders that had experience with mosquitoes).

**Figure 10.**
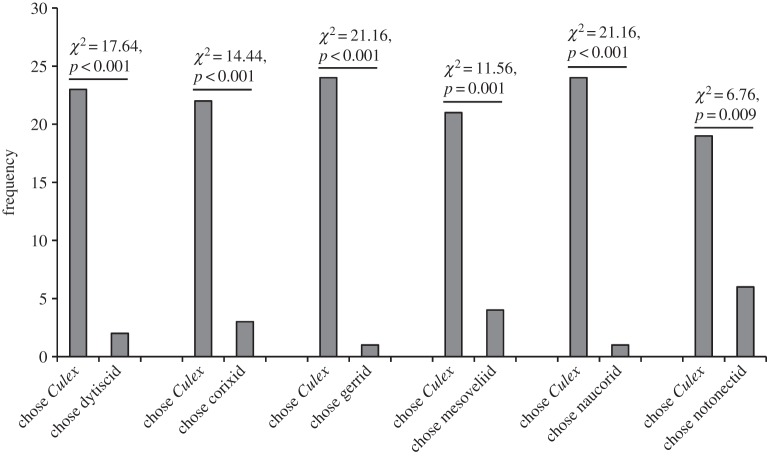
Results from simultaneous-presentation testing of *P*. *wanlessi* with larvae of *C*. *quinquefasciatus* and a variety of non-mosquito aquatic prey. All test spiders had been maintained on the standard terrestrial diet (no experience with mosquitoes or with any other aquatic prey; see table 2). Prey: lures presented to test spiders in water. *n*=25 for each pair of prey types.

Perhaps the strongest evidence that *P*. *wanlessi*'s preference for terrestrial and for aquatic mosquitoes is innate came from experiments in which spiders were tested with aquatic prey after being on the standard terrestrial diet (i.e. no experience with any aquatic prey). These spiders chose mosquito larvae significantly more often than they chose any of a variety of aquatic insects (figure 10). Moreover, when presented with a mosquito larva and a naucorid, data for spiders on the standard terrestrial diet were not significantly different from data for spiders on an alternative diet that included these particular prey (table 4, comparison *c*).

### 4.4 Which does *Paracyrba wanlessi* prefer, adult or juvenile mosquitoes?

**Figure 11.**
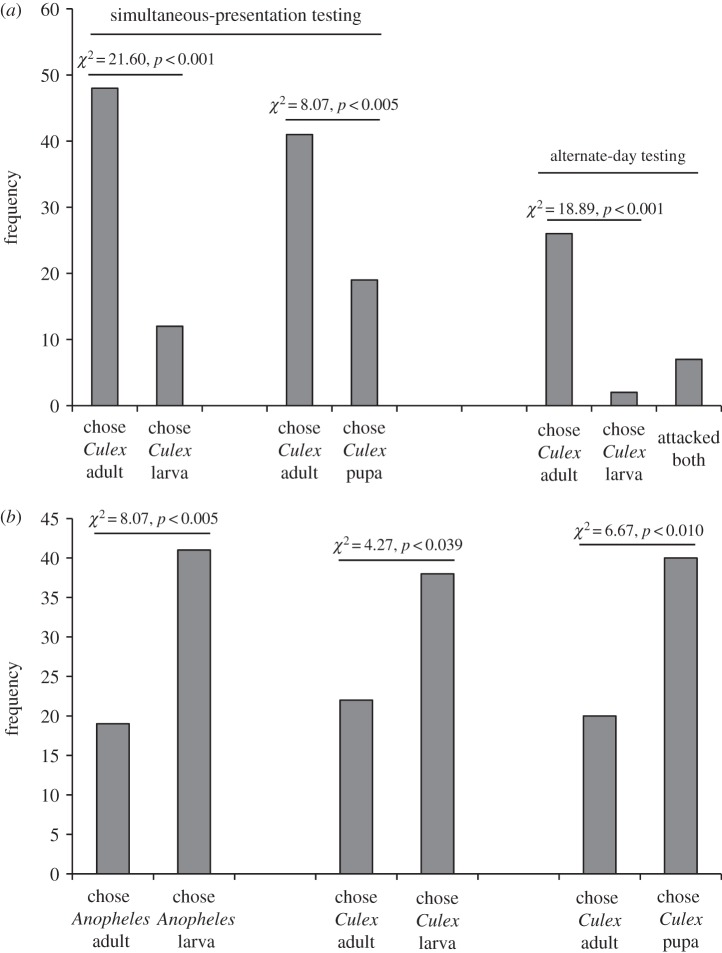
Results from testing *P*. *wanlessi* with two prey types: one, but not the other, being in its normal location (in or away from water). Test spiders had been maintained on the standard terrestrial diet (no experience with mosquitoes; see table 2). Prey: lures presented to test spiders. (*a*) *Culex* *quinquefasciatus* larvae or pupae paired with *C*. *quinquefasciatus* adult females. Both prey away from water. Simultaneous-presentation testing: *n*=60 for each pair of prey types. Alternate-day testing: *n*=35. (*b*) *Anopheles gambiae* larvae paired with *A. gambiae* adult females, and *C*. *quinquefasciatus* larvae or pupae paired with *C*. *quinquefasciatus* adult females. Both prey in water. Simultaneous-presentation testing: *n*=60 for each pair of prey types.

When test spiders that had been maintained on the standard terrestrial diet (i.e. spiders that had no experience with mosquitoes) were presented with two different mosquito prey simultaneously and one of these prey, but not the other, was in its normal location, significantly more spiders chose mosquito adults instead of larvae or pupae when both prey were away from water (figure 11*a*), but the reverse was the case when both prey were in water (significantly more chose larvae or pupae instead of adults; figure 11*b*). These findings suggest an interesting answer to the question of which prey *P*. *wanlessi* prefers—terrestrial mosquitoes (i.e. adults) or aquatic mosquitoes (i.e. larvae and pupae). Apparently, *P*. *wanlessi* attends simultaneously to mosquito stage (adult versus juvenile) and to mosquito location (away from versus in water). Mosquito stages in their normal location are preferred.

### 4.5 Which does *Paracyrba wanlessi* prefer, predatory sequences with prey in water or predatory sequences with prey away from water?

This question was considered by using alternate-day testing with lures in water on one day and lures away from water on the next or previous day (sequence determined at random). When using test spiders that had been maintained on the standard aquatic diet, the number that chose aquatic mosquitoes (larvae) in water was significantly more than the number that chose terrestrial mosquitoes (adults) away from water (table 5). We also found that, when using test spiders which had been maintained on the standard terrestrial diet, the number that chose pupae (aquatic mosquitoes) in water was significantly more than the number that chose adult mosquitoes away from water (a trend towards more test spiders choosing larvae (aquatic mosquitoes) in water than adults away from water was not significant, *p*=0.067). Moreover, when using test spiders that had been maintained on the standard terrestrial diet, the number that chose terrestrial mosquitoes (adults) in water was significantly more than the number that chose aquatic mosquitoes (larvae or pupae) away from water.

**Table 5.** Results from alternate-day testing of *P*. *wanlessi*maintained on different diets (*n*=45 for each row). Juvenile or adult mosquitoes presented to *P*. *wanlessi* in water on one day and the reverse (adult or juvenile mosquitoes, respectively) presented to *P*. *wanlessi* away from water on previous or following day (order decided at random). (Standard aquatic diet (no experience with mosquitoes): see table 3. Standard terrestrial diet (no experience with mosquitoes): see table 2.)

diet	prey 1 (in water)	prey 2 (away from water)	chose only prey 1	chose only prey 2	attacked both	McNemar test
standard aquatic diet	*Culex quinquefasciatus* larva	*Culexquinquefasciatus* adult	13	3	29	*χ*^2^=5.06, *p*=0.025
standard terrestrial diet	*Culexquinquefasciatus* larva	*Culexquinquefasciatus* adult	14	5	26	*χ*^2^=3.37, *p*=0.067
	*Culexquinquefasciatus* pupa	*Culexquinquefasciatus* adult	13	1	31	*χ*^2^=8.64, *p*=0.003
	*Culexquinquefasciatus* adult	*Culexquinquefasciatus* larva	35	1	9	*χ*^2^=30.25, *p*<0.001
	*Culexquinquefasciatus* adult	*Culexquinquefasciatus* pupa	38	2	5	*χ*^2^=30.63, *p*<0.001

**Figure 12.**
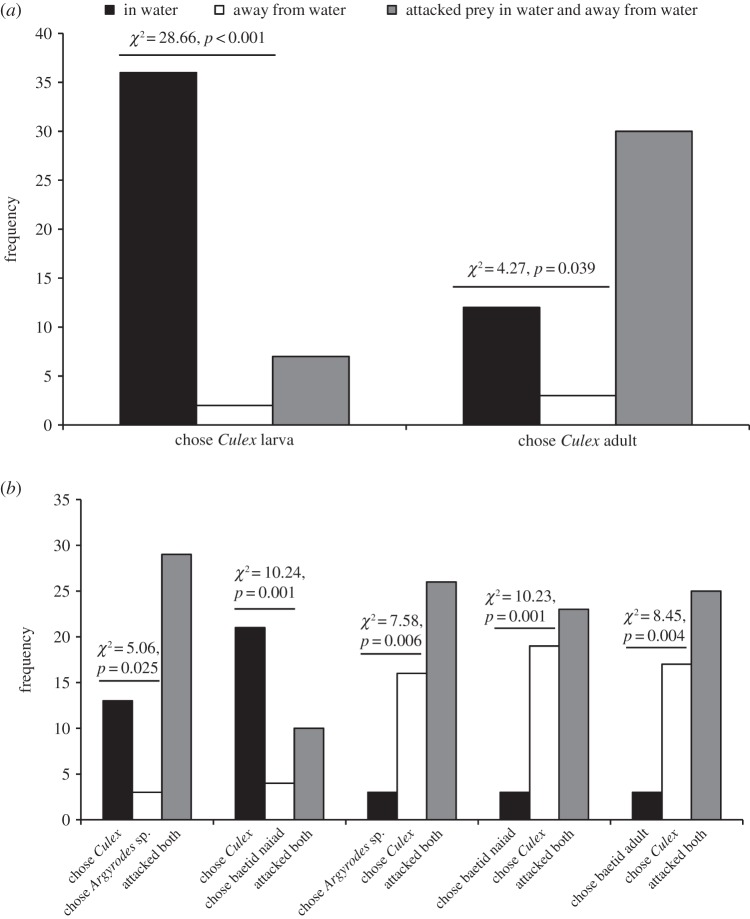
Results from alternate-day testing of *P*. *wanlessi* with prey that were presented in water on one day and away from water on the other day. All test spiders had been maintained on the standard terrestrial diet (no experience with mosquitoes, baetids or spiders; see table 2). (*a*) Larvae or adult females of *C*. *quinquefasciatus* (*n*=45 for each pair). (*b*) Adult females of *C*. *quinquefasciatus* and *Argyrodes* sp. or baetid naiads (*n*=45 for each pair of prey types, except that *n*=35 when baetid naiads were away from water).

When the prey in water and the prey away from water were both mosquito larvae or were both mosquito adults, test spiders that had been maintained on the standard terrestrial diet chose the prey that was in water significantly more often than they chose the prey that was away from water (figure 12*a*), and the number that chose mosquito adults in water was significantly more than the number that chose *Argyrodes* sp. or baetid naiads away from water (figure 12*b*). However, it was not simply that *P*. *wanlessi* always preferred prey in water. When the alternatives were adult mosquitoes away from water instead of *Argyrodes* sp., baetid naiads or baetid adults in water, significantly more chose the mosquitoes.

For *P*. *wanlessi*, the identity of the prey and whether the prey was in or away from water appeared to be two factors that acted together to influence prey-choice decisions. In §4.4 (figure 11), we showed that, when both prey were in water and both prey were mosquitoes, but one was an adult (terrestrial stage) and the other was a juvenile (aquatic stage), *P*. *wanlessi*'s preference was for the aquatic stage. However, preferences were reversed when both of these prey were away from water (i.e. the adults were preferred when both prey were away from water).

Congruence of an environmental factor (in water versus away from water) with prey type appeared to be a critical factor for *P*. *wanlessi*. We might characterize *P*. *wanlessi* as being a predator that prefers to encounter juvenile mosquitoes, an aquatic prey type, in water and as a predator that prefers to encounter adult mosquitoes, a terrestrial prey type, away from water. However, in §4.5, we showed that *P*. *wanlessi* also expressed a preference for encountering its mosquito prey in water instead of encountering its mosquito prey away from water (table 5 and figure 12*a*), even when the mosquitoes were adults (i.e. a terrestrial prey type). Yet, *P*. *wanlessi* expressed a preference for adult mosquitoes away from water when the alternatives were non-mosquito prey in water (figure 12*b*). These findings suggest that, when the choice is between the preferred prey type (a mosquito) in the non-preferred environment and a non-preferred prey (a non-mosquito prey) in the preferred environment, the prey-type preference is dominant.

## 5. General discussion

Ecologists and evolutionary biologists often use formal scientific taxonomy for specifying preferred prey when discussing the prey-choice behaviour and preferences of predators (e.g. [34]). We could be conventional and say that *E*. *culicivora* and *P*. *wanlessi* are two predators that prefer mosquitoes as prey, but this is too simplistic. For *E*. *culicivora*, but apparently not for *P*. *wanlessi*, the distinctions between adult male and adult female mosquitoes and between adult female mosquitoes that have or have not recently fed on blood are important [26]. Different distinctions are important for *P*. *wanlessi*, as this is a salticid with preferences pertaining to juvenile as well as to adult mosquitoes (table 6). For *P*. *wanlessi*, but not for *E. culicivora*, whether prey is in or away from water is relevant.

**Table 6.** Prey preferences of *P. wanlessi*. Prey are presented either away from water or in water. (P: prefers first choice over second choice. N: does not prefer first choice over second choice. X: not tested.)

			first choice							
			away from water				in water			
			mosquito adults	terrestrial prey	mosquito juveniles	aquatic prey	mosquito adults	terrestrial prey	mosquito juveniles	aquatic prey
second choice	away from water	mosquito adults	—	N	N	X	P	N	P	N
		terrestrial prey	P	—	X	X	P	X	X	X
		mosquito juveniles	P	X	—	X	P	X	P	X
		aquatic prey	X	X	X	—	P	X	X	X
	in water	mosquito adults	N	N	N	N	—	N	P	X
		terrestrial prey	P	X	X	X	P	—	X	X
		mosquito juveniles	N	X	N	X	N	X	—	N
		aquatic prey	P	X	X	X	X	X	P	—

Findings from experiments in which we varied the diets of test spiders imply that *P*. *wanlessi*'s preferences are innate, but these innate preferences appear to be unusually intricate. For people, the different active life stages of a mosquito (larva, pupa and adult) differ strikingly in appearance and habitat. For example, an adult mosquito has two wings, six legs, a proboscis and a pair of large compound eyes, whereas a juvenile mosquito is a wingless and legless insect that has no compound eyes and no proboscis. Yet, despite distinctive differences in morphology, appearance and location, *P*. *wanlessi* expresses preferences for all three of these mosquito life stages when the alternatives were non-mosquito prey. This is perhaps the most remarkable implication of the findings from our experiments.

People readily assign all three life stages to a category called ‘mosquito’, but only after learning about the mosquito's life cycle. People can also learn about formal scientific taxonomy and then assign mosquitoes as a group, regardless of the life stage (larva, pupa and adult), sex of the adults (male and female) and the prior diet of the adult females (blood and no blood) of the members of the group, to a particular insect family, ‘Culicidae’. It would be exceedingly far-fetched to suggest that *P*. *wanlessi* and *E*. *culicivora* use scientific taxonomy and understand mosquito life cycles. When we focus on *P*. *wanlessi*'s own classification systems, we get a distinctively different perspective. According to *P*. *wanlessi*'s own classification system, adult and juvenile mosquitoes are distinct prey categories because our experimental findings imply that *P*. *wanlessi* distinguishes between adult and juvenile mosquitoes, expressing a preference for adults away from water and juveniles in water.

When the underlying goal is to understand behaviour, cognition and adaptive specialization, including specialized perceptual, decision-making and prey-capture proficiencies, emphasis on formal scientific taxonomy can be particularly misleading. When we want to discuss a predator's behaviour and cognitive capacities, appreciating that a predator's own prey classification system can be very different from our own becomes essential. For discussing behaviour and cognition, the expressions ‘specialized’, ‘generalized’, ‘choice’ and ‘preference’ are appropriate and needed. If we are looking for the evidence needed for conclusions about behaviour and cognition, then data pertaining to diet simply will not suffice [8].

Curio [35] used the expression ‘predatory versatility’ for predators that deploy conditional predatory strategies. The most familiar conditional predatory strategies might come from predators that have an innate predisposition to deploy different prey-specific prey-capture tactics. However, conditional predatory strategies are known also to be based on the predator being innately predisposed to deploy different tactics when in different environmental settings. In *Portia*, a genus of spartaeine salticids, we find the most pronounced expression of predator versatility known for spiders. Each individual of *Portia* can, at different times, be a predator that captures prey by using its own self-built web, or a predator that hunts for prey completely away from webs, or a predator that invades the webs of other spiders where it preys on the resident spiders, the resident spider's eggs or other arthropods in the other spider's web, including ensnared insects as well as kleptoparasitic spider species that live in the other spider's web. Moreover, when away from webs, when in its own web and when in another spider's web, each *Portia* individual deploys a repertoire of different prey-specific prey-capture tactics, with each prey-specific tactic being an example of specialization on a different prey type [9]. For understanding *Portia*'s predatory strategy, we need to distinguish sharply between questions about specialization and questions about natural diet.

*Paracyrba* is another spartaeine genus and, like *Portia*, *P*. *wanlessi* is a versatile predator, but there is no evidence of specialization by *P*. *wanlessi* on spiders as prey. Our findings show instead that, on the basis of prey-capture behaviour, prey-choice behaviour and preferences, *P*. *wanlessi* is a mosquito specialist. Evidence of mosquito specialization has either been absent or highly limited in the many reports of predation on adult mosquitoes by other spiders, including other salticids, or in the reports of predation on juvenile mosquitoes by spiders from other families [36–53]. Instead of simply eating mosquitoes, *P*. *wanlessi* has a remarkable capacity to make decisions based on attending to the prey's identity (i.e. different mosquito stages) and location (at or away from water).
